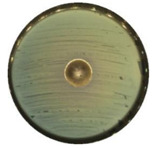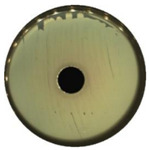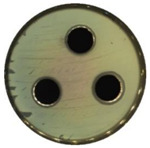# Hydrothermally Assisted Fabrication of TiO_2_-Fe_3_O_4_ Composite Materials and Their Antibacterial Activity

**DOI:** 10.3390/ma13214715

**Published:** 2020-10-22

**Authors:** Adam Kubiak, Marta Kubacka, Elżbieta Gabała, Anna Dobrowolska, Karol Synoradzki, Katarzyna Siwińska-Ciesielczyk, Katarzyna Czaczyk, Teofil Jesionowski

**Affiliations:** 1 Faculty of Chemical Technology, Institute of Chemical Technology and Engineering, Poznan University of Technology, Berdychowo 4, PL-60965 Poznan, Poland; adam.l.kubiak@doctorate.put.poznan.pl (A.K.); marta00146@wp.pl (M.K.); katarzyna.siwinska-ciesielczyk@put.poznan.pl (K.S.-C.); 2 National Research Institute, Institute of Plant Protection, Węgorka 20, PL-60318 Poznan, Poland; elzbieta.gabala@gmail.com; 3 Department of Biotechnology and Food Microbiology, Poznan University of Life Sciences, Wojska Polskiego 48, PL-60637 Poznan, Poland; anna.dobrowolska@up.poznan.pl (A.D.); katarzyna.czaczyk@up.poznan.pl (K.C.); 4 Institute of Molecular Physics, Polish Academy of Sciences, Smoluchowskiego 17, PL-60179 Poznan, Poland; karol.synoradzki@ifmpan.poznan.pl; 5 Institute of Low Temperature and Structure Research, Polish Academy of Sciences, Okólna 2, PL-50422 Wrocław, Poland

**Keywords:** titania, magnetite, hydrothermal method, antibacterial agents, reusability

## Abstract

The TiO_2_-Fe_3_O_4_ composite materials were fabricated via the hydrothermal-assisted technique. It was determined how the molar ratio of TiO_2_ to Fe_3_O_4_ influences the crystalline structure and morphology of the synthesized composite materials. The effect of the molar ratio of components on the antibacterial activity was also analyzed. On the basis of XRD patterns for the obtained titanium(IV) oxide-iron(II, III) oxide composites, the two separate crystalline forms—anatase and magnetite —were observed. Transmission electron microscopy revealed particles of cubic and tetragonal shape for TiO_2_ and spherical for Fe_3_O_4_. The results of low-temperature nitrogen sorption analysis indicated that an increase in the iron(II, III) oxide content leads to a decrease in the BET surface area. Moreover, the superparamagnetic properties of titanium(IV) oxide-iron(II, III) oxide composites should be noted. An important aim of the work was to determine the antibacterial activity of selected TiO_2_-Fe_3_O_4_ materials. For this purpose, two representative strains of bacteria, the Gram-negative *Escherichia coli* and Gram-positive *Staphylococcus aureus*, were used. The titanium(IV) oxide-iron(II, III) oxide composites demonstrated a large zone of growth inhibition for both Gram-positive and Gram-negative bacteria. Moreover, it was found that the analyzed materials can be reused as antibacterial agents in three consecutive cycles with good results.

## 1. Introduction

Currently, there is a significant increase in research into the design of new antimicrobial materials to control and reduce the number of microorganisms around us. Bacterial infections are a problem that affects millions of people worldwide every year. They raise both social and medical concerns [1,2]. Bacterial infections may appear in postoperative wounds if the procedure for the sterilization of the instruments and implants was not strictly followed [3]. Bacteria are capable of forming a biofilm structure, thus protecting themselves against the environment and the human immune system [4,5]. At the implant site, an infection develops, which spreads to the whole body and may cause life-threatening complications [6,7]. To reduce the likelihood of infection, many different antibiotics are administered to patients at high doses, which may cause bacterial mutations and increase their drug resistance [8,9]. Many compounds and materials with antibacterial properties have been developed to prevent bacterial infections, including quaternary ammonium compounds [10], carbon nanotubes [11], metal ions [12], metal oxide molecules [13], and precious metal-based materials [14]. However, these antibacterial agents have disadvantages including environmental pollution, complexity, and the high cost of the production process or substrates [8]. There is consequently a need to continue to develop effective antibacterial materials.

Among the many materials with antibacterial properties, titanium(IV) oxide deserves particular attention, in view of properties such as good chemical and thermal stability, as well as photocatalytic activity [15]. The first report on the biocidal properties of TiO_2_ was published in 1985 by Matsunaga et al. [16]. Subsequently, many articles have been published focusing on the inactivation of bacteria, viruses, and other pathogens, as well as cancer cells by photoactive titanium(IV) oxide [17,18,19]. However, the use of this material is subject to a practical limitation, namely the need to separate the material after the process and to perform its recovery [20]. Therefore, attention is paid to the synthesis of composite materials containing TiO_2_ and Fe_3_O_4_, which provide an opportunity to eliminate this problem. Iron(II, III) oxide is a good candidate, which, as a result of synthesis with titanium(IV) oxide by various techniques, significantly improves its properties. In addition, the material is magnetically recoverable and non-toxic and facilitates recycling after the process. 

Much research has been conducted in recent years on the synthesis of iron oxides, in particular iron(II, III) oxide, as is reflected in the number of published documents in databases such as Scopus (approximately 2000 search results in 2019). This results from the fact that this material is a common ferrite with a cubic inverse spinel structure, showing good electrical and magnetic properties [21]. Thus, it is used in magnetic data media, such as audio and video media [22,23], but also in medicine as contrast media for magnetic resonance imaging [24], magnetic hyperthermia [25], magnetic cell separation [26], as well as in medical diagnostics and cancer therapy [27,28]. In addition, Fe_3_O_4_ is widely used in synthesis processes with other oxides such as silica, zinc, or copper oxides, because the systems obtained have a wide range of applications. Systems based on iron(II, III) oxide and silica are used, among others, in the purification of aqueous media [29,30] and in medicine [31,32]. However, materials based on iron(II, III) oxide and zinc oxide are used for improved removal of elements from watercourses [33], as catalysts [34,35], and as antibacterial agents [35,36]. The above review of the existing literature shows clearly that magnetite-based composite materials have applications in a range of scientific fields.

The available scientific literature indicated that the TiO_2_-Fe_3_O_4_ systems can be used in many areas, including photocatalysis [37,38], removal of hazardous compounds from aqueous solutions [39,40], and magnetic resonance imaging [41]. A recoverable and reusable TiO_2_-Fe_3_O_4_ photocatalyst was fabricated via a one-step co-precipitation method by Arabzadeh et al. [42]. To obtain the mentioned photocatalyst, nanoparticles of commercial P25 were used as a source of titanium(IV) oxide. Moreover, the fabricated material was applied in photocatalytic degradation of tartrazine. Babudurai et al. [43] used the anatase nanoparticles for the synthesis of TiO_2_-Fe_3_O_4_ nanocomposite via the co-precipitation method. The obtained system was used as a photoactive material in the degradation process of Orange G. Salamat et al. [44], who obtained magnetic core-shell Fe_3_O_4_@TiO_2_ nanoparticles by the two step hydrothermal method, which additionally were calcined at 400 °C. The Researchers proved that the core-shell Fe_3_O_4_@TiO_2_ based materials showed high photooxidation activity in the degradation of the organic pollutant from steel industry wastewater. The core-shell materials were also synthesized by Kermani et al. [45]. The mentioned TiO_2_@Fe_3_O_4_ magnetic materials were applied as a catalyst in the ozonation of catechol. The synthesis of titanium(IV) oxide-iron(II, III) oxide composites was also reported by Yuxiang et al. [46], who used the two step sol-gel method assisted by the calcination process to obtain superparamagnetic photocatalysts. Li et al. [47] indicated that the addition of graphene oxide to TiO_2_-Fe_3_O_4_ materials improved their photocatalytic properties in Vis light. Bui et al. [48] described the modification of TiO_2_-Fe_3_O_4_, with magnesium amino-functionalized clay via the sol-gel method. Additionally, the authors used the obtained materials for the water treatment process realized by applying photo-Fenton and photocatalytic reactions. Beketova et al. [49] reported the application of titanium(IV) oxide nanotubes decorated with iron(II, III) oxide nanoparticles via the co-precipitation and/or solvothermal methods. The fabricated materials were used as photocatalysts in the degradation process of methylene blue. 

Based on the literature review, it should be noted that the synthesis of TiO_2_-Fe_3_O_4_ composites is gaining importance, due to the good photocatalytic properties of titanium(IV) oxide and the magnetic properties of iron(II, III) oxide. However, attention should be paid to the fact that, based on the review mentioned above, the titanium(IV) oxide-iron(II, III) oxide products were mainly fabricated by the co-precipitation and sol-gel methods. Moreover, many researchers used commercial titanium(IV) oxide, e.g., anatase nanoparticles and P25. Additionally, in many cases, the synthesis of the mentioned materials was multistage and assisted by the calcination process. Therefore, we decided to apply the one step hydrothermally-assisted method to obtain TiO_2_-Fe_3_O_4_ materials with a well-formed crystalline structure and morphology. What is more important is that it consumes much lower energy than conventional methods and does not generate toxic waste; thanks to that, it can be an element of a strategy toward environmentally friendly production. Furthermore, although in the scientific literature, the antibacterial activities for titanium(IV) oxide and iron(II, III) oxide separately were shown, the antibacterial properties for TiO_2_-Fe_3_O_4_ composites have not been widely described until now. For this reason, in this work, the antibacterial activity of the selected TiO_2_-Fe_3_O_4_ composites was determined. For this purpose, two representative strains of bacteria, the Gram-negative *Escherichia coli* and Gram-positive *Staphylococcus aureus*, were used. Moreover, for the analyzed composite materials, the reusability in three consecutive cycles was determined.

## 2. Materials and Methods

### 2.1. Materials

TiCl_4_ (97%), FeCl_2_∙4H_2_O (98%), FeCl_3_∙6H_2_O (97%) NH_3_∙H_2_O (25%), and (CH_3_)_2_CHOH (99.5%, IPA) were purchased from Sigma-Aldrich (St. Louis, MO, USA). The reagents used were analytical grade. Moreover, deionized water was used in all experiments. 

### 2.2. Synthesis of TiO_2_-Fe_3_O_4_ Composites

In a typical one step hydrothermally-assisted synthesis procedure, 1.5 g of FeCl_2_∙4H_2_O and 3 g of FeCl_3_∙6H_2_O (in the molar ratio Fe^2+^:Fe^3+^ = 1:2) were dissolved in 100 cm^3^ of deionized water. In the next step, to the obtained solution of iron(II, III) oxide precursor, a specified amount of a 10% aqueous solution of TiCl_4_ was added. The TiO_2_:Fe_3_O_4_ molar ratio was controlled by holding the volume of iron salts constant and varying the volume of titania precursor. The mixture was then placed on a magnetic stirrer (Ika, Werke, Staufen, Germany), and an ammonia solution was added. After the addition of a few drops of ammonia, the solution had a grey-black color. The final pH was 9–10. Then, the resulting mixture was subjected to hydrothermal treatment at 200 °C for 12 h. The obtained titanium(IV) oxide-iron(II, III) oxide composites were separated with the use of an external magnetic field and washed with deionized water. Finally, the TiO_2_-Fe_3_O_4_ materials were dried at 45 °C for 12 h and underwent classification. For comparison, titanium(IV) oxide and iron(II, III) oxide samples were fabricated as reference samples.

### 2.3. Characterization of Synthesized Composites

The dispersion properties, e.g., the particle size distribution (PSD) of the synthesized composite, were analyzed using the non-invasive backscattering method applying a Zetasizer Nano ZS apparatus (Malvern Instruments Ltd., Malvern, UK).

The morphology and microstructure of the synthesized composite were investigated using an EVO40 scanning electron microscope (SEM) (Zeiss, Jena, Germany) and a Hitachi HT7700 transmission electron microscope (TEM) (Hitachi, Tokyo, Japan) operating in High-Contrast (HC) and High-Resolution (HR) modes. 

To determine the crystalline structure of the fabricated products, the X-ray diffraction method was applied. The Rigaku Miniflex 600 apparatus (Rigaku, Tokyo, Japan) operating with Cu Kα radiation (λ = 1.5418 Å) was used. The patterns were obtained over an angular range of 10–80°. The diffraction patterns were evaluated by the Rietveld method using the Fullprof software [50]. The crystallite size of the synthesized composites in the vertical direction to the corresponding lattice plane was determined using Scherrer’s equation [29,30] with the constant equal to 0.891. Quantitative analysis, including phase composition with the standard deviation, was calculated using the Reference Intensity Ratio (RIR) method from the most intensive independent peak of each phase.

The low-temperature nitrogen sorption allowed determining the textural properties such as: surface area, pore volume, and pore diameter. In the above-mentioned analysis, the apparatus ASAP 2020 porosimeter (Micromeritics Instrument Co., Norcross, GA, USA) was used. Before measurement, the materials were degassed at 120 °C for 4 h. The surface area was determined by the multipoint BET method using adsorption data in the relative pressure *(p/p_0_)* range of 0.05–0.30.

The SQUID magnetometer (MPMS-XL, Quantum Design, San Diego, CA, USA) was used to perform magnetic measurements. The temperature dependences of the magnetization were measured in a magnetic field of 0.1 T in the temperature range of 2–300 K. Magnetization loops were collected at 5 and 300 K in magnetic fields up to 5 T.

In order to identify the functional groups present on the surface of the composite materials, Fourier transform infrared spectroscopy was carried out. The FTIR spectra were measured over a wavenumber range of 4000–420 cm^−1^ using a Vertex 70 apparatus (Bruker, Leipzig, Germany).

The Jupiter STA 449 F3 apparatus (Netzsch GmbH, Bad Berneck im Fichtelgebirge, Germany) was applied to determine thermogravimetric curves. The analysis was performed under flowing nitrogen at a heating rate of 10 °C/min in a temperature range of 30–1000 °C.

### 2.4. Antibacterial Activity

Antibacterial tests were carried out using two methods: the agar diffusion method [51] and the standard shake flask method [52]. Both tests were performed using Gram-negative *Escherichia coli* (ATCC 10536) and Gram-positive *Staphylococcus aureus* (ATCC 33592).

For the first of the above-mentioned methods (agar diffusion), the microorganisms were grown in nutrient broth (OXOID CM 0001). The broth contained (in g/dm^3^) meat extract 1.0, yeast extract 2.0, peptone 5.0, sodium chloride 5.0, glucose 20.0 and agar 15.0, respectively. The final pH of the mixture was 7.4 ± 0.2. Moreover, the cultures of bacteria were grown at 35 ± 2 °C for 24 h. To determine the antimicrobial properties, the Muller–Hinton medium (OXOID CM 0337) was applied. This medium contained (in g/dm^3^) dehydrated beef infusion 300.0, casein hydrolysate 17.5, starch 1.5, and agar 17.0 (the final pH is 7.3 ± 0.1). The plates were inoculated using indicator microorganisms in the range of 10^7^ CFU/cm^3^ (100 µL) and stayed for 15 min for adsorbing the microorganisms to the surface. Next, the wells (14 mm diameter) were cut in agar plates, and 0.1 g of analyzed composite: (7)TiO_2_-(3)Fe_3_O_4_, (5)TiO_2_-(5)Fe_3_O_4_, (3)TiO_2_-(7)Fe_3_O_4_, were placed into the wells. Then, the plates were incubated at 35 °C ± 2 °C for 24 h. After this time, the diameter of the inhibitory zone surrounding the discs was measured in mm. Tetracycline discs (6 mm, containing 30 µg of antibiotic) and reference samples (TiO_2_ and Fe_3_O_4_) were applied as control samples. 

For the second technique (shake flask method), frozen beads of the examined species were thawed, subcultured onto nutrient broth (OXOID CM 0001), and incubated at 35 ± 2 °C for 24 h. Cultures were centrifuged at 4500 *g* for 10 min, and cells were washed in deionized water. Cultures were resuspended in water and adjusted to 0.5 on the McFarland scale (1.5 × 10^8^ CFU/cm^3^) by the McFraland Densitometer (Biosan). For the experiments, the final concentration of bacteria was adjusted to the level of 5.0 × 10^7^ CFU/cm^3^. The composite materials—(7)TiO_2_-(3)Fe_3_O_4_, (3)TiO_2_-(7)Fe_3_O_4_—were sterilized by autoclaving (121 °C, 15 min), and next, zero-point-one grams of analyzed sterile oxide materials were incubated with 100 cm^3^ of bacterial suspensions at 37 °C and 230 rpm. The cell density of the suspensions before introducing the material and after 30, 60 and 120 min in contact with the material were determined using the pour plate method. These suspensions were decimally diluted in sterile physiological saline, plated on plate count agar, and incubated at 35 °C ± 2 °C for 24–48 h to determine the number of surviving bacteria. The materials after use were recovered with an external magnetic field and used a second and third time, to determine the stability of the antibacterial activity. The procedure for the determination of the reduction of the viability of the analyzed materials was the same as in the case of the first use. 

## 3. Results and Discussion

### 3.1. Dispersion and Morphology 

For any material, irrespective of its type, the dispersion properties, as well as morphology and microstructure are significant factors that affect its potential applications. In the first step, for the fabricated samples, the Particle Size Distributions (PDS) were determined and the SEM analysis was performed (Table 1). 

For the reference sample, a TiO_2_ maximum volume contribution of 25.3% corresponds to agglomerates of 1106 nm in diameter. Additionally, a Polydispersity Index (PdI) of 0.142 was determined for titanium(IV) oxide. The particle size distribution of iron(II, III) oxide confirmed the presence of particles in the range of 105 to 295 nm. The maximum volume contribution (27.9%) came from particles of 164 nm in diameter. This sample had a polydispersity index equal to 0.312. Based on the presented results of the particle size distribution, it is shown that the molar ratio of TiO_2_:Fe_3_O_4_ has a meaningful influence on the dispersion properties. The sample (7)TiO_2_-(3)Fe_3_O_4_ obtained with a molar ratio of TiO_2_:Fe_3_O_4_ = 7:3 contains particles in the range of 255–825 nm. The maximum volume contribution of 26.6% corresponds to agglomerates of 458 nm in diameter. Moreover, the PdI of this sample is 0.211, which indicated that the composite material is homogeneous. In the case of the sample synthesized with the equimolar ratio of TiO_2_:Fe_3_O_4_, it contains particles in the range of 190–955 nm. The maximum volume contribution of 18.2% corresponds to agglomerates of 458 nm in diameter. Furthermore, for the material mentioned above, the PdI = 0.256 was determined; whereas, slightly smaller particles were observed in the sample (3)TiO_2_-(7)Fe_3_O_4_, as confirmed by their diameter range of 164–531 nm and by the dominant diameter (295.3 nm). It should be noted that the analyzed composite is relatively homogeneous, as indicated by the low value of the polydispersity index (0.289). Based on the dispersion analysis, it was confirmed that increasing the content of Fe_3_O_4_ in the synthesized composites leads to products with smaller particles. Moreover, it should be noted that all binary materials have a monomodal particle size distribution, as well as a high tendency to agglomerate.

**Figure 1:**
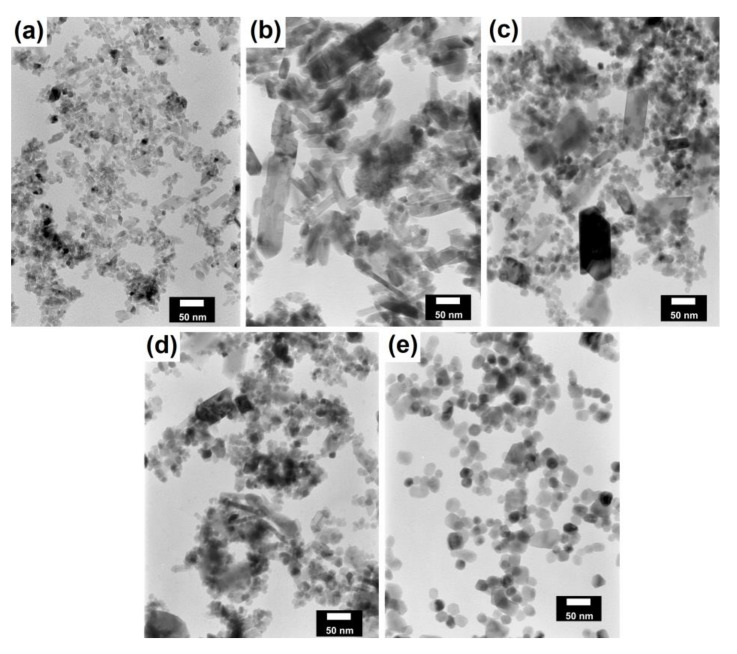
TEM images for composite materials and reference samples: (**a**) TiO_2_; (**b**) (7)TiO_2_–(3)Fe_3_O_4_; (**c**) (5)TiO_2_–(5)Fe_3_O_4_; (**d**) (3)TiO_2_–(7)Fe_3_O_4_; (**e**) Fe_3_O_4._

In the next step of physicochemical characterization, scanning electron microscopy was applied to evaluate the morphology of the synthesized TiO_2_-Fe_3_O_4_ systems. The presented SEM pictures indicate the effect of the molar ratio of the components on the morphology of the products. Moreover, it was shown that for all analyzed materials, the tendency to agglomerate is observed. However, increasing the contribution of iron(II, III) oxide causes smaller aggregates. The results obtained by scanning electron microscopy correspond with the dispersion analysis. Furthermore, to determine the shape of the TiO_2_ and Fe_3_O_4_ phases, TEM analysis was carried out (Figure 1).

In the TEM images for TiO_2_ and Fe_3_O_4_, we can observe typical nanoparticles (diameter <50 nm), being in agreement with the theory of Karatutlu and co-authors [53], on the formation of nanoparticles in the liquid phase. Titanium(IV) oxide particles of a cubic and tetragonal shape with a diameter of about 25 nm are shown. For iron(II, III) oxide, a spherical particle shape with a diameter in the range of 10–20 nm was observed. Subsequently, the titanium(IV) oxide-iron(II, III) oxide composites were examined. For all of the synthesized composites, the same shape of particles as for the reference samples was observed. Moreover, particles with a rod-like structure were also noted. The presence of the structure mentioned above can be associated with impurities or the unreacted substrate during synthesis, e.g., FeOOH. For the precise characterization of composite materials, HR-TEM and mapping by EDS were carried out. Both above-mentioned analyses were performed for selected samples: (7)TiO_2_-(3)Fe_3_O_4_ and (3)TiO_2_-(7)Fe_3_O_4_.

**Figure 2:**
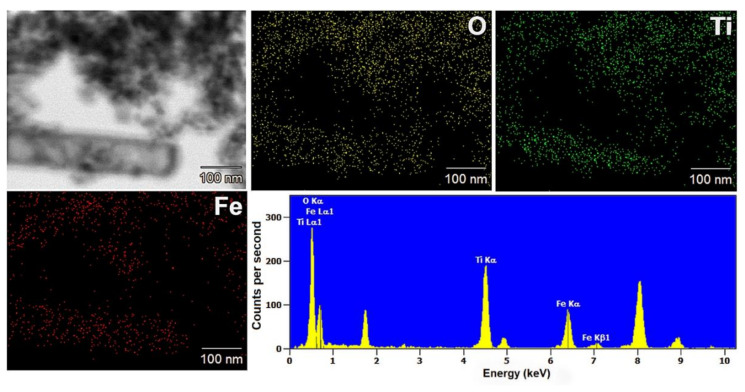
Results of EDS mapping for the (7)TiO_2_-(3)Fe_3_O_4_ composite.

**Figure 3:**
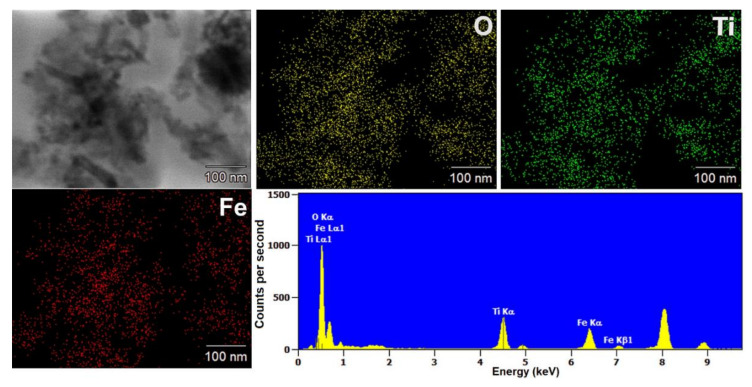
Results of EDS mapping for the (3)TiO_2_-(7)Fe_3_O_4_ composite.

EDS mapping enabled the determination of the distribution of the analyzed elements (titanium, oxygen, and iron) in the synthesized composite materials. The EDS maps of the selected materials are shown in Figure 2 and Figure 3.

Based on the obtained results, it is indicated that the distribution of titanium(IV) oxide and iron(II, III) oxide is not homogeneous for the analyzed composites. Furthermore, both analyzed elements: titanium and iron are in larger aggregates of nanoparticles. However, it should be noted that the localization of titanium and iron elements may indicate the occurrence of surface interactions between components in composite systems.

**Figure 4:**
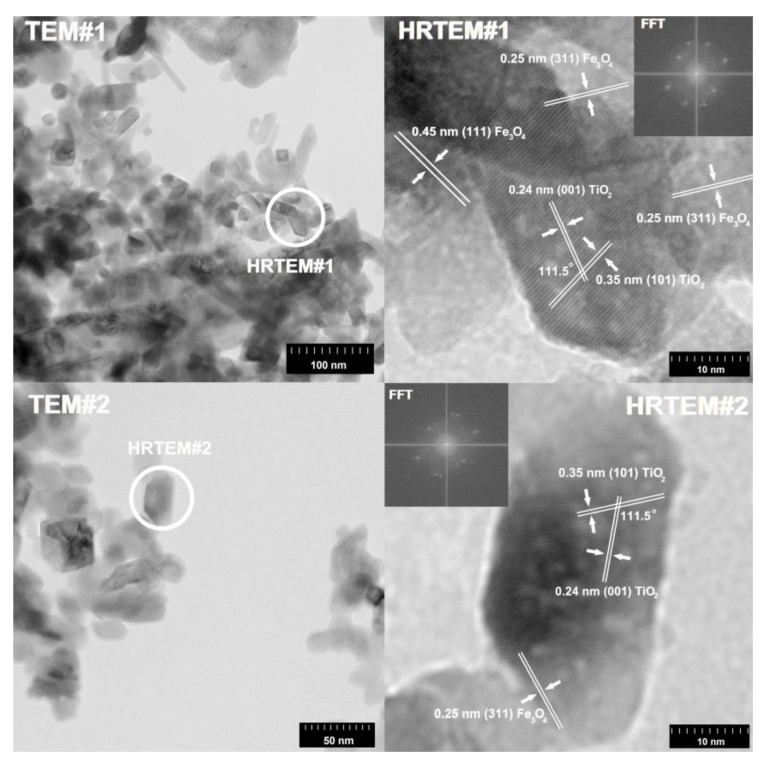
TEM, HR-TEM, and FFT results for the (7)TiO_2_-(3)Fe_3_O_4_ sample.

**Figure 5:**
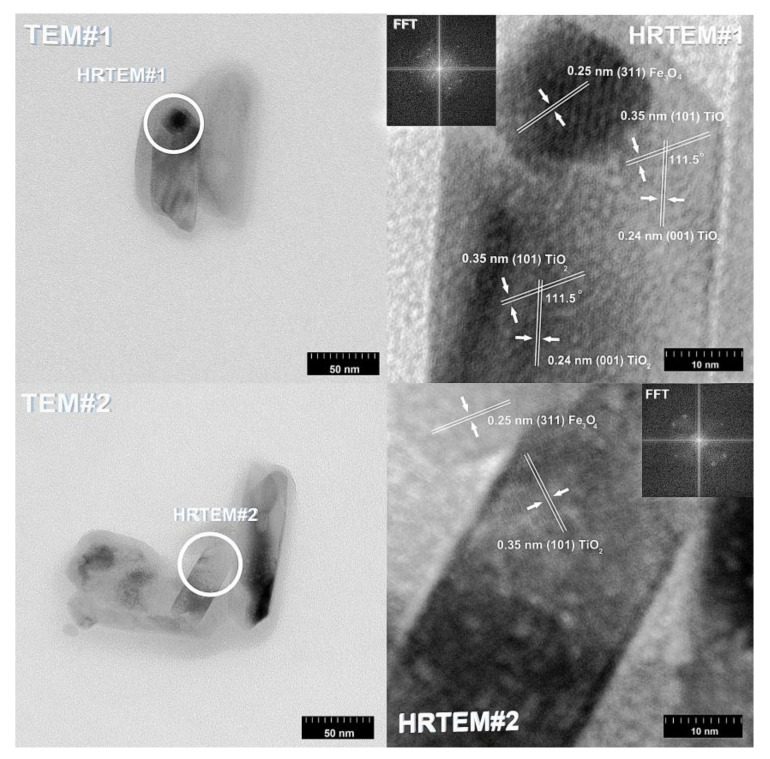
TEM, HR-TEM, and FFT results for the (3)TiO_2_-(7)Fe_3_O_4_ sample.

Transmission electron microscopy in high-resolution mode was used to determine crystallographic spacings and planes. The results of HR-TEM are shown in Figure 4 and Figure 5.

According to previously reported results for the (7)TiO_2_-(3)Fe_3_O_4_ composite, the crystallographic spacings of 0.24 nm and 0.35 nm, characteristic of the anatase phase, were observed [54]. Moreover, the angle in the crystallographic lattice is 111.5°. In Figure 5, the crystallographic lattice parameters characteristic for magnetite are noted. Spacings equal to 0.24 nm and 0.45 nm characteristic for planes (111) and (311) in the Fe_3_O_4_ phase were observed [55]. Very similar results were obtained for the second analyzed titanium(IV) oxide-iron(II, III) oxide composite, (3)TiO_2_-(7)Fe_3_O_4_. On this basis, it was concluded that regardless of the TiO_2_:Fe_3_O_4_ molar ratio, these materials exhibit the same crystalline lattice parameters as indicated by the HR-TEM analysis presented above. However, the interactions between iron(II, III) oxide and titanium(IV) oxide should also be described. Based on available scientific knowledge, it was concluded that the incorporation of magnetite particles on titania involves only surface junctions, because we did not observe a mixed crystalline structure, for example ilmenite (FeTiO_3_) or others. It should be noted, however, that the main objective of the presented research is not to fabricate a mixed crystalline structure of titania and iron oxide, but composites with a stable junction between these two components, which was confirmed by the conducted EDS analyses: coverage of EDS maps for titanium and iron. Furthermore, attention should be paid to the fact that both TiO_2_ and Fe_3_O_4_ occur in the form of aggregated nanoparticles; therefore, the surface-junction is possible, because both phases (TiO_2_ and Fe_3_O_4_) surround each other, which was observed in the presented TEM and HR-TEM images.

### 3.2. Crystalline Structure

**Figure 6:**
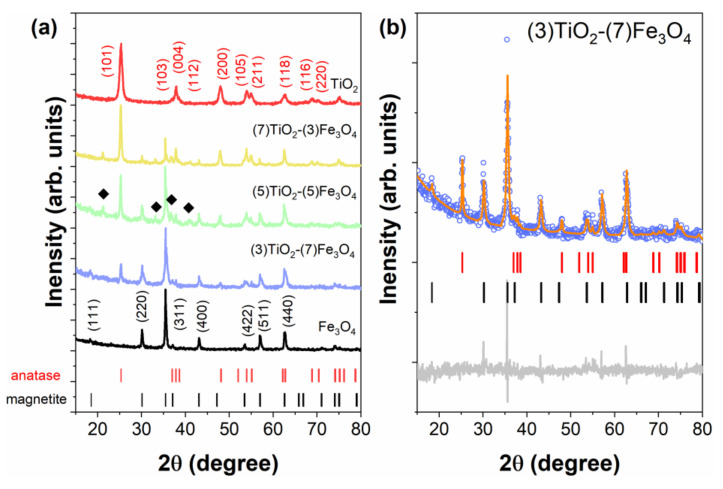
XRD patterns for all studied TiO_2_-Fe_3_O_4_ composites and reference samples (**a**). Peaks marked by diamonds belong to the α-FeOOH impurity phases. For the most pronounced peaks, Miller indices are given. (**b**) XRD pattern (open circles) and Rietveld refinement (solid orange line) for the selected (3)TiO_2_-(7)Fe_3_O_4_ composite sample. The difference curve of the experimental and calculated intensity is shown at the bottom. The upper and lower ticks represent Bragg positions corresponding to the anatase and magnetite phases, respectively.

The XRD analysis was performed to examine whether the obtained materials have a defined crystal structure. The patterns of the synthesized composite materials and reference samples are presented in Figure 6a. For all samples, a set of distinct reflections indicates the highly crystalline nature of the obtained materials. Moreover, the observed peaks can be assigned to the anatase phase (space group *I*4_1_/*amd*, No. 141) and/or magnetite Fe_3_O_4_ (space group *Fd*3*m*, No. 227) phases. For TiO_2_, the following crystallographic planes were determined: (101), (103), (104), (112), (200), (105), (211), (204), (116) (220), (215). The planes determined for magnetite were as follows: (111), (220), (311), (222), (400), (422), (333), (440), (622). The intensity of the mentioned peaks changes successively as the content of samples changes. For some composite samples (e.g., (5)TiO_2_-(5)Fe_3_O_4_), we observed a few additional peaks (marked with diamonds in Figure 6a) originating from the α-FeOOH impurity phase (space group *Pbnm*, No. 62). 

The mass fraction and lattice parameters values for each phase were determined according to Rietveld refinement and are summarized in Table 2. An exemplary Rietveld refinement of the XRD pattern recorded at room temperature for the (3)TiO_2_-(7)Fe_3_O_4_ sample is shown in Figure 6b. The calculated phase composition is in good agreement with the nominal one. It should also be noted that the lattice parameters for the composite materials and reference samples are comparable and fit well to data already published for similar systems [54,55].

The average crystallite size (D) characteristic for the anatase and magnetite phases was calculated using Scherrer’s equation (*D* = *K*λ/(*B*cosθ)) and the obtained results are presented in Table 2. The average size of the crystallites is around ~25 nm, for both the TiO_2_ and Fe_3_O_4_ phases, in almost all samples. Only for pure TiO_2_ are the crystallites smaller (*D*~15 nm). The crystallite values are smaller than the particle size determined by TEM measurements, as the particles may consist of many crystallites. 

Based on previous work by members of the research team, such as Jędrzak et al. [56], who synthesized superparamagnetic nanoparticles of Fe_3_O_4_, and Siwińska-Ciesielczyk et al. [57,58], who fabricated titania nanoparticles using different methods, it was attempted to obtain TiO_2_-Fe_3_O_4_ materials with well-defined crystallinity using a hydrothermally-assisted method. In the literature reports, among others, Tan et al. [59] described the application of Fe_3_O_4_@TiO_2_ materials in the sorption of uranium. However, because of the good sorption properties of the materials used (high surface area), the crystalline structure was not well formed. Khashan et al. [60] synthesized Fe_3_O_4_@TiO_2_ nanoparticles via the co-precipitation method. The crystalline structure of the described materials indicated a well-formed anatase structure, but only single diffraction peaks derived from Fe_3_O_4_. A different crystalline structure was described by Zhu et al. [61], who synthesized Fe_3_O_4_/TiO_2_ nanoparticles via a three step process. The crystallinity of the obtained materials showed diffraction bands derived from anatase, rutile, and cubic magnetite. Of course, many other researchers have described similar magnetic materials with a core-shell structure, e.g., Chen et al. [62].

### 3.3. Parameters of the Porous Structure

**Figure 7:**
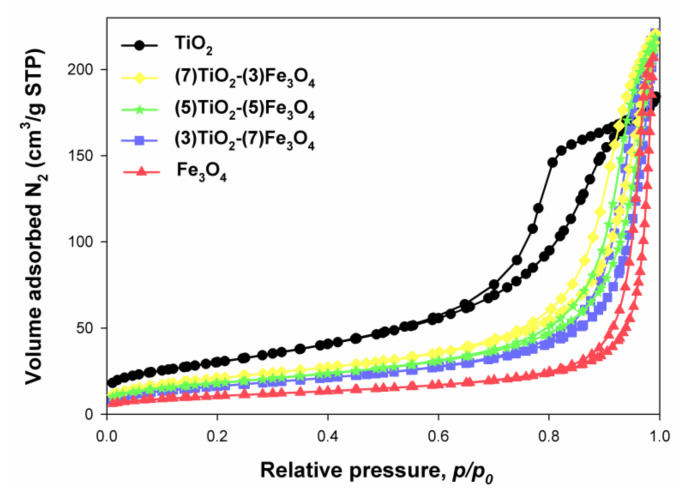
N_2_ adsorption/desorption isotherms of TiO_2_-Fe_3_O_4_ composites and reference samples.

To determine the parameters of the porous structure of the fabricated composites, the low-temperature nitrogen sorption was carried out. The obtained results are presented in Table 3 and in Figure 7, respectively.

The reference sample of titanium(IV) oxide had the higher BET surface area among the studied materials (107 m^2^/g). Moreover, the pore volume and pore diameter for TiO_2_ were respectively 0.284 cm^3^/g and 9 nm. The iron(II, III) oxide was found to have a BET surface area of 37 m^2^/g, a pore volume of 0.317 cm^3^/g, and a pore diameter of 34 nm. The (7)TiO_2_-(3)Fe_3_O_4_ sample had a BET surface area of 75 m^2^/g, with a pore diameter and pore volume of 18 nm and 0.337 cm^3^/g. For the titanium(IV) oxide-iron(II, III) oxide composite obtained at an equimolar ratio, the values were A_BET_ = 65 m^2^/g, V_p_ = 0.335 cm^3^/g, and S_p_ = 19 nm. For the system with a molar ratio TiO_2_:Fe_3_O_4_ = 3:7, the BET surface area was 59 m^2^/g, the pore diameter 21 nm, and the pore volume 0.334 cm^3^/g. 

Comparing the results obtained for TiO_2_-Fe_3_O_4_ materials with the available literature, we should take note of the study by Feizpoor et al. [63], who obtained a TiO_2_-Fe_3_O_4_ system by heating at the boiling point under a reflux condenser. For the material with a molar ratio TiO_2_:Fe_3_O_4_ = 4:1, a BET surface area of 17 m^2^/g was obtained, with a pore diameter of 49.2 nm and a pore volume of 0.2 cm^3^/g. Shojei et al. [64] synthesized materials based on titania and magnetite using the sol-gel method. The resulting system had a surface area of 160 m^2^/g and a pore volume of 0.34 cm^3^/g. Li et al. [65], who synthesized a material containing TiO_2_ and Fe_3_O_4_ by an alkaline hydrothermal etching-assisted crystallization method, reported the BET surface area of the obtained system to be 187 m^2^/g. Shojaie et al. [66] obtained TiO_2_-Fe_3_O_4_-Ag ternary systems by an ultrasonic method supported by a hydrothermal method. For a sample containing 0.5 g of TiO_2_, 0.05 g of Fe_3_O_4_, and 2 g of Ag, the BET surface area was 45 m^2^/g, the pore volume 0.3 cm^3^/g, and the pore diameter 14.02 nm. Fisli et al. [67] synthesized TiO_2_-Fe_3_O_4_ materials using heteroagglomeration. For a material with a molar ratio TiO_2_:Fe_3_O_4_ = 1:0.5, the BET surface area was 91 m^2^/g. However, for samples obtained with the ratio of titanium(IV) oxide to iron(II, III) oxide equal to 1:1 and 1:2, the respective BET surface areas were 64 m^2^/g and 57 m^2^/g.

### 3.4. Magnetic Properties

**Figure 8:**
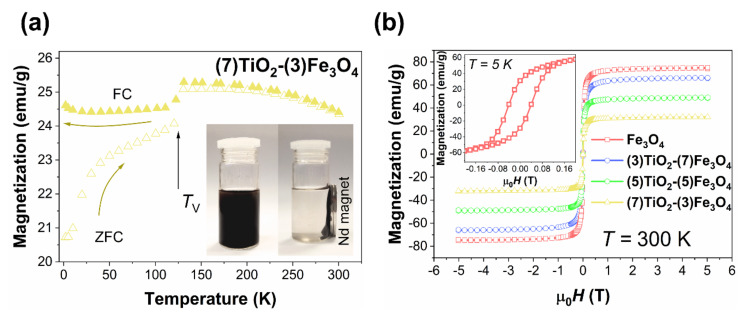
Magnetic properties of TiO_2_-Fe_3_O_4_ composites: (**a**) Magnetization of selected composite vs. temperature. The inset shows magnetic separation of TiO_2_-Fe_3_O_4_ composites suspended in water. (**b**) Magnetization vs. external magnetic field. The inset shows the magnetic loop for pristine Fe_3_O_4_ at 5 K.

All magnetic properties of titanium(IV) oxide-iron(II, III) oxide composites are summarized in Figure 8. As inferred from this figure, all prepared materials revealed similar magnetic properties as pure nano-Fe_3_O_4_. The temperature dependence of magnetization (Figure 8a) shows a strong bifurcation of the Zero-Field-Cooling (ZFC) [68] curve and Field-Cooled (FC) curve. A kink in both the ZFC and FC curves at ~120 K is related to the Verwey transition [69]. The temperature at which this structural transition occurs (*T*_V_) is stoichiometry [70], shape [71], and size dependent [72]. As the addition of the TiO_2_ does not change the *T*_V_, we conclude that the size and chemical composition of magnetite particles are conserved, which manifests the high quality of our samples. In *M*(*T*) relationships, there is no clear trace of blocking temperature (*T*_B_), which usually manifests itself as a wide maximum in the ZFC curve. Part (b) of the Figure 8 shows magnetization loops. At RT, there is no sign of magnetic hysteresis and remanence. However, at 5 K (inset of Figure 8b), we observe a clear hysteresis loop for all magnetite-based materials. The magnetization saturation value (*M*_s_) of pure Fe_3_O_4_ is 73.7(5) emu/g, and it is smaller than for bulk magnetite, which may result from spin disorder, variations in crystallinity, or antiphase domain boundaries [73]. With the addition of TiO_2_ [74], which is paramagnetic, the *M*_s_ value drops. All results indicate superparamagnetic-like behavior for all samples containing Fe_3_O_4_. However, the absence of a clear anomaly for the blocking phenomenon in *M*(*T*) curves and the relatively large size of Fe_3_O_4_ particles (>25/50 nm) could suggest that our materials are rather the ferromagnetic monodomain than superparamagnetic [75,76]. Nevertheless, at RT, all prepared composite materials can be easily separated from water solution by a permanent magnet, as is demonstrated in the inset of Figure 8a.

### 3.5. FTIR Spectroscopy

**Figure 9:**
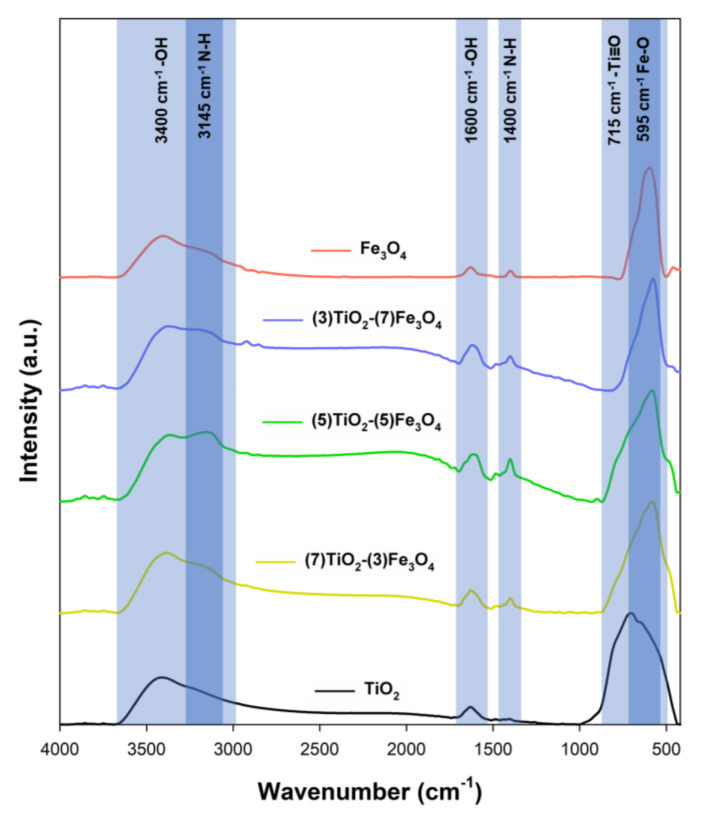
FTIR spectra for the TiO_2_, Fe_3_O_4_, and TiO_2_-Fe_3_O_4_ materials.

In order to identify the functional groups present on the surface of the composite materials and reference samples, Fourier transform infrared spectroscopy was carried out. The FTIR spectra for the reference samples and TiO_2_-Fe_3_O_4_ materials are shown in Figure 9.

In the FTIR spectrum for the TiO_2_ reference sample, the stretching vibrations of the –Ti≡O group (715 cm^−1^) [77] were observed. For the Fe_3_O_4_ reference sample, the band occurring at 595 cm^−1^ corresponds to stretching vibrations of Fe–O [9]. Furthermore, for all fabricated materials, the stretching (3400 cm^−1^) [78] and bending (1600 cm^−1^) [79] vibrations corresponding to the hydroxyl group (–OH) were noted. The FTIR spectra for the TiO_2_-Fe_3_O_4_ materials contained bands characteristic for both TiO_2_ and Fe_3_O_4_, as well as additional bands for the stretching (3145 cm^−1^) and bending (1400 cm^−1^) vibrations of the N–H groups, derived from ammonia, which was the pH regulator in the synthesis. Moreover, it should be noted that the TiO_2_ and Fe_3_O_4_ bands are very close on the FTIR spectra; for this reason, the Ti = O band for the sample (3)TiO_2_-(7)Fe_3_O_4_ is not visible.

### 3.6. Thermal Analysis

**Figure 10:**
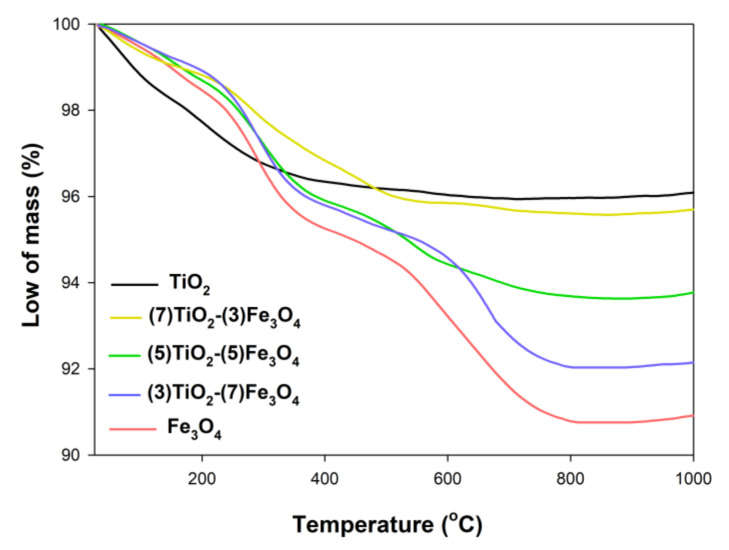
TGA curves for the TiO_2_-Fe_3_O_4_ materials and reference samples.

Thermal stability is a significant physicochemical parameter, which can enable the potential use of composite materials. The thermal stability of the synthesized materials was evaluated using TGA analysis (Figure 10). 

The total decrease in mass for the titanium(IV) oxide sample was 3.8%, which was bound up with the elimination of bound surface H_2_O (in a temperature range of 0–400 °C). Similar observations were made by Chan et al. [80], who observed a total decrease in mass for a sample equal to 20% with the removal of bound surface water in the temperature range of 0–300 °C. Three mass decreases were observed for the Fe_3_O_4_ reference sample, in the temperature ranges of 0–250 °C (2.5%), 300–600 °C (2.5%), and 600–800 °C (1.4%), attributed respectively to the evaporation of water and to phase transformations from magnetite to maghemite and from maghemite to hematite. Jędrzak et al. [81] obtained particles of magnetite, which also exhibited good thermal stability; the total weight loss for that material was approximately 10%. For the (7)TiO_2_-(3)Fe_3_O_4_ composite, the TGA curve is similar to that observed for the TiO_2_ reference sample, with the total mass decrease in this case amounting to 4.5%. In the case of products fabricated with molar ratios of TiO_2_:Fe_3_O_4_ = 5:5 and 3:7, three mass decreases were observed, resulting from the removal of surface water, the phase transitions from magnetite to maghemite and maghemite to hematite, where the total weight loss for these materials was 5.4% and 7.9%, respectively. All analyzed systems had good thermal stability up to 1000 °C, which is in accordance with the available scientific reports [43].

### 3.7. Antibacterial Properties

The antibacterial activity of (7)TiO_2_-(3)Fe_3_O_4_, (5)TiO_2_-(5)Fe_3_O_4_, and (3)TiO_2_-(7)Fe_3_O_4_ materials was evaluated against representative strains of bacteria, Gram-negative *Escherichia coli*, and Gram-positive *Staphylococcus aureus.* The results of antimicrobial activity testing using the agar diffusion method are shown in Table 4 and Table 5. The analyzed materials (7)TiO_2_-(3)Fe_3_O_4_, (5)TiO_2_-(5)Fe_3_O_4_, and (3)TiO_2_-(7)Fe_3_O_4_ indicated a high zone of inhibition, which proved their good antibacterial activity against Gram-positive *Staphylococcus aureus* (≥ 21.0 mm) in the absence of the activity of oxides (TiO_2,_ Fe_3_O_4_) and the weak action of tetracycline (positive control). Lesser activity of the tested composites was observed in the case of *Escherichia coli* (zone of inhibition between 15 and 18 mm) with high effectiveness of the antibiotic against this bacteria.

**Figure 11:**
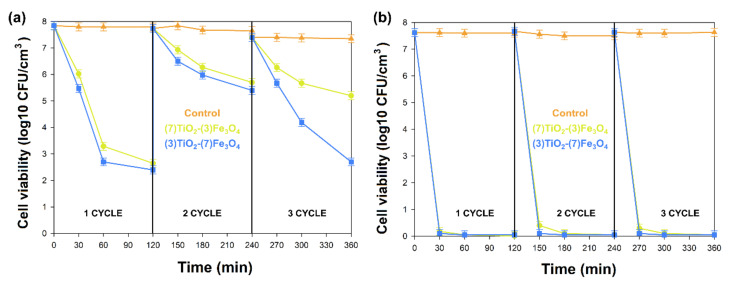
Antibacterial activity of examined materials during three cycles against *Staphylococcus aureus* (**a**) and *Escherichia coli* (**b**).

To check the antimicrobial activity of the tested materials in subsequent applications, (7)TiO_2_-(3)Fe_3_O_4_ and (3)TiO_2_-(7)Fe_3_O_4_ composites were selected, and the shake flask method was used. During the experiments (120 min, three cycles), the number of bacteria cells in solution without any material was monitored (control). The tested material showed high antibacterial activity against both examined strains. Samples (7)TiO_2_-(3)Fe_3_O_4_ and (3)TiO_2_-(7)Fe_3_O_4_ destroyed the Gram-negative *Escherichia coli* in 30 min and kept these properties during the second and third use (Figure 11). The use of (7)TiO_2_-(3)Fe_3_O_4_ and (3)TiO_2_-(7)Fe_3_O_4_ against Gram-positive *Staphylococcus aureus* caused the reduction of the number of bacteria for about 1, 4, and 5 log cycles after 30, 60, and 120 min of the experiment, respectively. The second and third use of the analyzed materials against *Staphylococcus aureus* indicated that they were still active. During the second use of the analyzed materials, the reduction of the amounts of bacteria was at the level of two log cycles. The material (3)TiO_2_-(7)Fe_3_O_4_ used for the third time had similar properties as when it was used for the first time. 

The antibacterial activity of metal oxides and their hybrids, including TiO_2_ and Fe_3_O_4_ nanoparticles, has been the subject of much scientific research [82,83,84]. The creation of the structure TiO_2_/Fe_3_O_4_ may enhance these properties, especially when activated by different forms of light [85,86,87]. Furthermore, the heterostructures TiO_2_/Fe_3_O_4_ with other compounds (mostly gold or silver compounds) gave positive results in terms of antimicrobial growth action [88,89]. The antimicrobial effect of such a composite is connected with lipid peroxidation due to the interaction between the membrane and reactive oxygen species (ROS) [89]. The succession of this killing mechanism is the degradation of the cell wall and cytoplasmic membrane, leading to the leakage of cellular contents and cell lysis [90].

The antimicrobial effect of the (7)TiO_2_-(3)Fe_3_O_4_, (5)TiO_2_-(5)Fe_3_O_4_, and (3)TiO_2_-(7)Fe_3_O_4_ materials was investigated with Gram-negative and Gram-positive, commonly used for the examination of the antibacterial activity of various materials. Two methods of testing the antibacterial properties were used in these investigations: the agar diffusion method and the standard shake flask method. In both methods, the tested materials indicated antibacterial activity against Gram-negative *Escherichia coli* and Gram-positive *Staphylococcus aureus* (Table 4 and Table 5 and Figure 11). It is well known that the destruction of the outer membrane and cell wall of bacteria is crucial for bacterial cell death. The Gram-negative and Gram-positive bacteria have many differences in the structure of the membranes and cell wall. *Escherichia coli* has an outermost lipopolysaccharide layer, a thinner peptidoglycan layer (15–20 nm, in comparison to *Staphylococcus aureus* 20–80 nm), and a phospholipid bilayer (monolayer in *Staphylococcus aureus*) [91,92,93]. The different antibacterial mechanism of Fe_3_O_4_-TiO_2_ nanosheets against *Escherichia coli* and *Staphylococcus aureus* was also investigated by Ma et al. [87] using SEM analysis. Before the experiments, *Escherichia coli* exhibited intact and smooth membranes and a normal elongated morphology, but already after 15 min of exposure, the cells became deformed. However, for *Staphylococcus aureus*, no obvious cell wall and membrane destruction was noticed during the whole time of the investigations, and the cell shape changed from a regular sphere to an irregular shape. Furthermore, *Staphylococcus aureus* had a smaller diameter compared with *Escherichia coli,* and its cell surface was too small to be covered by the examined nanoparticles. 

The particularly important results obtained in our investigations are connected with the second and third use of the nanoparticles of (7)TiO_2_-(3)Fe_3_O_4_ and (3)TiO_2_-(7)Fe_3_O_4_ and the absence of the decrease in their antibacterial activity. These properties of the obtained particles have potential applications for the environment, the biomedical field, pharmaceuticals, and other commercial productions.

## 4. Conclusions

Based on the experience of the research team, hydrothermally-assisted synthesis was used to obtain TiO_2_-Fe_3_O_4_ composite materials. In a typical one-step hydrothermal synthesis procedure, highly crystalline materials were obtained. Based on the morphological analysis, the rod-like and cubic particle shape for titanium(IV) oxide and spherical shape for iron(II, III) oxide were observed. Additionally, all of the prepared composite samples exhibited superparamagnetic-like behavior. 

It should be noted that the proposed hydrothermally-assisted method allowed synthesizing the TiO_2_-Fe_3_O_4_ systems, which exhibited good antibacterial activity against *Staphylococcus aureus* and *Escherichia coli*. A key element of the study was the testing of the reusability of titanium(IV) oxide-iron(II, III) oxide composites as antibacterial agents. These experiments indicated the retention of good antibacterial activity over three successive cycles. Furthermore, the obtained TiO_2_-Fe_3_O_4_ materials demonstrated similar or better antibacterial activity compared with the reference titanium(IV) oxide and iron(II, III) oxide samples. A possible mechanism of the antibacterial action is the destruction of the outer membrane and cell wall of the bacteria, which leads to the death of the bacterial cell. The results of the tests of antibacterial activity indicated that the synthesized TiO_2_-Fe_3_O_4_ composite can find applications for the environment, the biomedical field, pharmaceuticals, and other commercial productions.

## Figures and Tables

**Table 1:** PSDs and SEM images of the obtained composite and references samples.

Sample	Particle Size Distributions (PSD)	SEM Images
TiO_2_	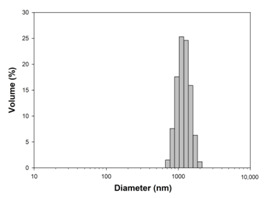	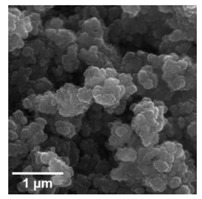
(7)TiO_2_-(3)Fe_3_O_4_	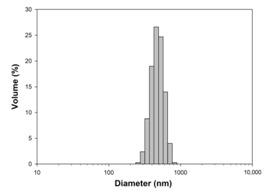	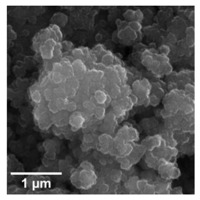
(5)TiO_2_-(5)Fe_3_O_4_	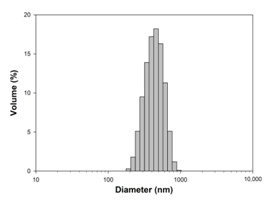	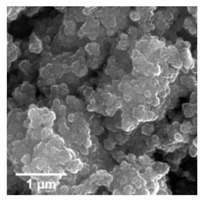
(3)TiO_2_-(7)Fe_3_O_4_	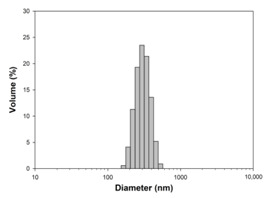	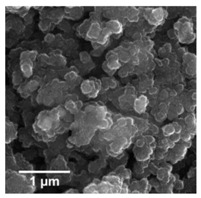
Fe_3_O_4_	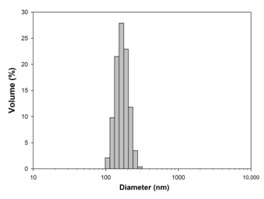	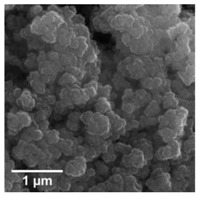

**Table 2:** Lattice parameters, phase composition, and average crystalline size for TiO_2_-Fe_3_O_4_ composite materials.

Sample	Lattice Parameters			Phase Composition (wt.%)		*D* (nm)	
	Anatase		Magnetite	Anatase	Magnetite	Anatase	Magnetite
	*a* (Å)	*c* (Å)	*a* (Å)				
TiO_2_	3.7937(7)	9.510(2)	-	100	-	15.3(1)	-
(7)TiO_2_-(3)Fe_3_O_4_	3.796(1)	9.513(2)	8.395(2)	79(2)	21(1)	24.1(2)	23.3(5)
(5)TiO_2_-(5)Fe_3_O_4_	3.800(1)	9.519(6)	8.382(1)	42(2)	58(2)	25.4(1)	24.3(2)
(3)TiO_2_-(7)Fe_3_O_4_	3.794(2)	9.508(8)	8.373(2)	19(1)	81(2)	24.9(3)	25.4(4)
Fe_3_O_4_	-	-	8.3883(6)	-	100	-	26.1(1)

**Table 3:** Parameters of the porous structure for the obtained TiO_2_-Fe_3_O_4_ materials and reference samples.

Sample	A_BET_ (m^2^/g)	V_p_ (cm^3^/g)	S_p_ (nm)
TiO_2_	107	0.284	9
(7)TiO_2_-(3)Fe_3_O_4_	75	0.337	18
(5)TiO_2_-(5)Fe_3_O_4_	65	0.335	19
(3)TiO_2_-(7)Fe_3_O_4_	59	0.334	21
Fe_3_O_4_	37	0.317	34

**Table 4:** The zone of inhibition of the tested TiO_2_-Fe_3_O_4_ composite materials and control samples.

Sample	Zone of Inhibition (mm)	
	*S. aureus*	*E. coli*
TiO_2_	0	0
Fe_3_O_4_	0	0
(7)TiO_2_-(3)Fe_3_O_4_	21.33 (±0.58)	15.33 (±0.58)
(5)TiO_2_-(5)Fe_3_O_4_	21.00 (±0.0)	15.67 (±0.58)
(3)TiO_2_-(7)Fe_3_O_4_	23.00 (±0.0)	17.67 (±0.58)
tetracycline	6.33 (±0.29)	24.67 (±0.58)

**Table 5:** Zone of inhibition for *Staphylococcus aureus* (a) and *Escherichia coli* (b): agar diffusion method.

	TiO_2_	Fe_3_O_4_	(7)TiO_2_-(3)Fe_3_O_4_—Top, (5)TiO_2_-(5)Fe_3_O_4_—Bottom Left, (3)TiO_2_-(7)Fe_3_O_4_—Bottom Right
**(a)**	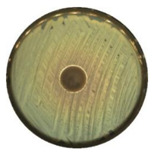	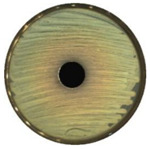	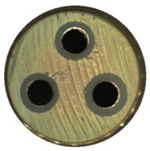
**(b)**